# Complete genome sequence of *Paraburkholderia* sp. strain 22B1P capable of utilizing 3-chlorobenzoate as a carbon source

**DOI:** 10.1128/mra.01235-23

**Published:** 2024-03-15

**Authors:** Ryota Moriuchi, Rion Sano, Shuma Fujii, Yuito Suzuki, Miyune Makita, Yo Kawashima, Takumi Shirakawa, Renki Shindo, Tatsumi Shinkai, Kaede Miura, Moka Hirose, Momiji Nakajima, Asahi Kurokawa, Rituparna Chetia, Chiharu Hirokawa, Tomoko Suzuki, Yukiko Ito, Hiroki Murano, Hideo Dohra, Naoto Ogawa, Yu Kanesaki

**Affiliations:** 1 Shizuoka Instrumental Analysis Center, Shizuoka University, Suruga-ku, Shizuoka, Japan; 2 Division of Technical Service, Shizuoka University, Suruga-ku, Shizuoka, Japan; 3 Shizuoka Prefectural Fujinomiya-Kita High School, Fujinomiya, Shizuoka, Japan; 4 OISCA Hamamatsu Kokusai High School, Hamamatsu, Shizuoka, Japan; 5 Shizuoka Prefectural Shimada Senior High School, Shimada, Shizuoka, Japan; 6 Hamamatsu Gakugei High School, Hamamatsu, Shizuoka, Japan; 7 Shizuoka Prefectural Shizuoka Higashi High School, Aoi-ku, Shizuoka, Japan; 8 Fuji Sacred Heart School, Susono, Shizuoka, Japan; 9 Faculty of Agriculture, Shizuoka University, Suruga-ku, Shizuoka, Japan; 10 Graduate School of Integrated Science and Technology, Shizuoka University, Suruga-ku, Shizuoka, Japan; 11 Research Institute of Green Science and Technology, Shizuoka University, Suruga-ku, Shizuoka, Japan; Montana State University, Bozeman, Montana, USA

**Keywords:** *Paraburkholderia*, 3-chlorobenzoate, complete genome, chlorocatechol, integrative and conjugative element, bioremediation

## Abstract

*Paraburkholderia* sp. strain 22B1P utilizes 3-chlorobenzoate as a carbon source. Complete genome sequencing of strain 22B1P revealed two chromosomes and two plasmids. The genes involved in the conversion of 3-chlorobenzoate to 3-chlorocatechol and those involved in the conversion of 3-chlorocatechol to 3-oxoadipate were located on chromosomes 2 and 1, respectively.

## ANNOUNCEMENT

Members of the genus *Paraburkholderia* (formerly *Burkholderia*) are known to degrade a variety of aromatic compounds, including chlorinated aromatic compounds such as polychlorinated biphenyls (1–3). Strain 22B1P was isolated from a soil sample in Yahatayama Park (34.9696N, 138.4004E), Shizuoka City, Japan, by screening based on the enrichment culture method using 3-chlorobenzoate (3-CB) (4, 5). Work in our laboratory (Ogawa, N., unpublished data) indicates that 22B1P can grow on 3-CB as a carbon and energy source. In this study, the complete genome sequence of *Paraburkholderia* sp. strain 22B1P was determined to expand our understanding of the ability of bacteria to degrade chlorinated aromatic compounds.

A single colony of strain 22B1P was grown in a glass flask with 20 mL of basal salts medium (4) containing 5 mM 3-CB at 27°C with shaking at 150 rpm for 4 days. Genomic DNA was extracted using the DNeasy Blood and Tissue Kit (QIAGEN) for subsequent sequencing on the Sequel IIe (Pacific Biosciences) and the NovaSeq 6000 (Illumina) sequencing platforms at Macrogen, Inc. (Seoul, Republic of Korea). Each library was constructed using the SMRTbell Express Template Prep Kit 3.0 (Pacific Biosciences) and the TruSeq DNA PCR-free Library Preparation Kit (Illumina), respectively. PacBio HiFi reads were filtered (read length, ≥6,000 bp) using BamTools v.2.5.1 (6), and the resulting reads, consisting of 52,773 reads with a maximum read length of 30,203 bp and an *N*_50_ value of 10,152 bp (total of 520,650,204 bp, with 52-fold genome coverage), were used for *de novo* assembly using Flye v.2.8.3 (7) with --plasmids option. Illumina short reads were used after cleaning with Trimmomatic v.0.39 (8) (read length, ≥100 bp; quality score, ≥15). The assembled genome was polished using Pilon v.1.23 (9) with trimmed Illumina reads (26,640,876 read pairs totaling 7,952,824,875 bp, with 801-fold genome coverage) obtained from 2 × 151 bp paired-end read sequencing. Genome annotation was performed using DFAST-core v.1.2.18 (10) with an in-house database containing 320 genome sequences of the genus *Paraburkholderia* deposited in the NCBI (National Center for Biotechnology Information) RefSeq database (17 August 2023). GeneMarkS2 v.1.14_1.25 (11) and MetaGeneAnnotator version 2008/08/19 (12), RNAmmer v.1.2 (13), and tRNAscan-SE v.2.0.5 (14) were used to identify protein-coding sequences, rRNA genes, and tRNA genes, respectively. The circular replicons were reoriented to begin with the *dnaA* gene for chromosome 1 and the replication initiation protein gene for other replicons, using Circlator v.1.5.3 (15). Default parameters were used for all software unless otherwise noted.

**Fig 1:**
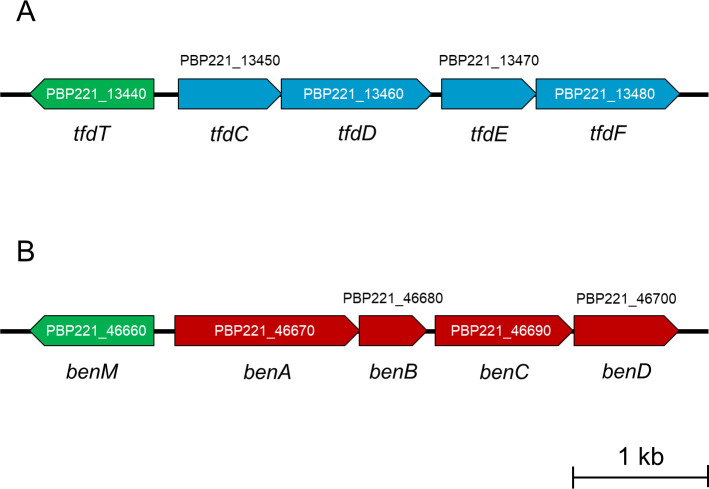
Gene clusters involved in the conversion of (**A**) 3-chlorocatechol and (**B**) 3-chlorobenzoate. Genes *tfdC*, *tfdD*, *tfdE*, *tfdF*, *benA*, *benB*, *benC,* and *benD* encode chlorocatechol 1,2-dioxygenase, muconate cycloisomerase, dienelactone hydrolase, maleylacetate reductase, benzoate 1,2-dioxygenase large subunit, benzoate 1,2-dioxygenase small subunit, benzoate 1,2-dioxygenase electron transfer component, and 1,6-dihydroxycyclohexa-2,4-diene-1-carboxylate dehydrogenase, respectively. The *tfdT* and *benM* genes encode LysR-type transcriptional regulators and could be involved in the regulation of expression of *tfd* and *ben* genes, respectively.

The genome of 22B1P comprised two circular chromosomes and two circular plasmids with a total genome size of 9,922,990 bp (Table 1). The genes involved in the conversion of 3-CB to 3-chlorocatechol (*ben* genes) (Fig. 1) were located on chromosome 2. Interestingly, the genes involved in the conversion of 3-chlorocatechol to 3-oxoadipate (*tfd* genes) (Fig. 1) were located on a putative integrative and conjugative element (16) on chromosome 1 (locus tag: PBP221_13280 to PBP221_16140), suggesting that the *tfd* genes were acquired by horizontal gene transfer. The highest average nucleotide identity score of strain 22B1P was 92.93% when compared with *Paraburkholderia terrae* DSM 17804^T^ (accession number GCA_002902925.1), suggesting that strain 22B1P is a novel species in the genus *Paraburkholderia*.

**TABLE 1:** General genomic features of *Paraburkholderia* sp. 22B1P

Replicon	Length (bp)	GC content (%)	No. of coding sequences	No. of rRNAs	No. of tRNAs	DDBJ/ENA/GenBank accession no.
Chromosome 1	3,784,668	62.9	3,305	12	55	AP028996
Chromosome 2	3,049,510	61.9	2,656	6	7	AP028997
Plasmid p22B1P1	2,481,578	61.9	2,180	0	2	AP028998
Plasmid p22B1P2	607,234	58.8	561	0	0	AP028999
Total	9,922,990	62.1	8,702	18	64	

## Data Availability

The complete genome sequence of *Paraburkholderia* sp. strain 22B1P has been deposited in DDBJ/ENA/GenBank under the accession numbers AP028996 to AP028999, and the raw sequence reads have been deposited under the accession numbers DRR506547 and DRR506548.

## References

[1] Lee Y, Lee Y, Jeon CO. 2019. Biodegradation of naphthalene, BTEX, and aliphatic hydrocarbons by Paraburkholderia aromaticivorans BN5 isolated from petroleum-contaminated soil. Sci Rep 9:860. doi:10.1038/s41598-018-36165-x 30696831 PMC6351602

[2] Bako CM, Mattes TE, Marek RF, Hornbuckle KC, Schnoor JL. 2021. Biodegradation of PCB congeners by Paraburkholderia xenovorans LB400 in presence and absence of sediment during lab bioreactor experiments. Environ Pollut 271:116364. doi:10.1016/j.envpol.2020.116364 33412450 PMC8183161

[3] Chain PSG, Denef VJ, Konstantinidis KT, Vergez LM, Agulló L, Reyes VL, Hauser L, Córdova M, Gómez L, González M, et al.. 2006. Burkholderia xenovorans LB400 harbors a multi-replicon, 9.73-Mbp genome shaped for versatility. Proc Natl Acad Sci U S A 103:15280–15287. doi:10.1073/pnas.0606924103 17030797 PMC1622818

[4] Ogawa N, Miyashita K. 1995. Recombination of a 3-chlorobenzoate catabolic plasmid from Alcaligenes eutrophus NH9 mediated by direct repeat elements. Appl Environ Microbiol 61:3788–3795. doi:10.1128/aem.61.11.3788-3795.1995 8526487 PMC167680

[5] Moriuchi R, Dohra H, Kanesaki Y, Ogawa N. 2021. Transcriptome differences between Cupriavidus necator NH9 grown with 3-chlorobenzoate and that grown with benzoate. Biosci Biotechnol Biochem 85:1546–1561. doi:10.1093/bbb/zbab044 33720310

[6] Barnett DW, Garrison EK, Quinlan AR, Strömberg MP, Marth GT. 2011. Bamtools: a C++ API and toolkit for analyzing and managing BAM files. Bioinformatics 27:1691–1692. doi:10.1093/bioinformatics/btr174 21493652 PMC3106182

[7] Kolmogorov M, Yuan J, Lin Y, Pevzner PA. 2019. Assembly of long, error-prone reads using repeat graphs. Nat Biotechnol 37:540–546. doi:10.1038/s41587-019-0072-8 30936562

[8] Bolger AM, Lohse M, Usadel B. 2014. Trimmomatic: a flexible trimmer for illumina sequence data. Bioinformatics 30:2114–2120. doi:10.1093/bioinformatics/btu170 24695404 PMC4103590

[9] Walker BJ, Abeel T, Shea T, Priest M, Abouelliel A, Sakthikumar S, Cuomo CA, Zeng Q, Wortman J, Young SK, Earl AM. 2014. Pilon: an integrated tool for comprehensive microbial variant detection and genome assembly improvement. PLoS One 9:e112963. doi:10.1371/journal.pone.0112963 25409509 PMC4237348

[10] Tanizawa Y, Fujisawa T, Nakamura Y. 2018. DFAST: a flexible prokaryotic genome annotation pipeline for faster genome publication. Bioinformatics 34:1037–1039. doi:10.1093/bioinformatics/btx713 29106469 PMC5860143

[11] Lomsadze A, Gemayel K, Tang S, Borodovsky M. 2018. Modeling leaderless transcription and atypical genes results in more accurate gene prediction in prokaryotes. Genome Res 28:1079–1089. doi:10.1101/gr.230615.117 29773659 PMC6028130

[12] Noguchi H, Taniguchi T, Itoh T. 2008. MetaGeneAnnotator: detecting species-specific patterns of ribosomal binding site for precise gene prediction in anonymous prokaryotic and phage genomes. DNA Res 15:387–396. doi:10.1093/dnares/dsn027 18940874 PMC2608843

[13] Lagesen K, Hallin P, Rødland EA, Staerfeldt HH, Rognes T, Ussery DW. 2007. RNAmmer: consistent and rapid annotation of ribosomal RNA genes. Nucleic Acids Res 35:3100–3108. doi:10.1093/nar/gkm160 17452365 PMC1888812

[14] Chan PP, Lin BY, Mak AJ, Lowe TM. 2021. tRNAscan-SE 2.0: improved detection and functional classification of transfer RNA genes. Nucleic Acids Res 49:9077–9096. doi:10.1093/nar/gkab688 34417604 PMC8450103

[15] Hunt M, Silva ND, Otto TD, Parkhill J, Keane JA, Harris SR. 2015. Circlator: automated circularization of genome assemblies using long sequencing reads. Genome Biol 16:294. doi:10.1186/s13059-015-0849-0 26714481 PMC4699355

[16] Delavat F, Miyazaki R, Carraro N, Pradervand N, van der Meer JR. 2017. The hidden life of integrative and conjugative elements. FEMS Microbiol Rev 41:512–537. doi:10.1093/femsre/fux008 28369623 PMC5812530

